# Reversible Intrapulmonary Vascular Dilatation in a Patient Treated With Luspatercept for Myelodysplastic Syndrome

**DOI:** 10.1016/j.chest.2026.02.012

**Published:** 2026-08-10

**Authors:** Fatema Al Lawati, Samir Gupta, Marie E. Faughnan, John Granton, Troy Climans, Amar Thakrar, Geneviève C. Digby

**Affiliations:** a Division of Respirology and Sleep Medicine, Department of Medicine, Queen’s University, Kingston, ON, Canada; b Division of Respirology, Department of Medicine, Li Ka Shing Knowledge Institute and St. Michael’s Hospital, Toronto, ON, Canada; c Division of Respirology, Department of Medicine, University Health Network, University of Toronto, Toronto, ON, Canada; d Division of Hematology, Department of Medicine Queen’s University, Queen’s University, Kingston, ON, Canada; e Division of Cardiology, Department of Medicine, Queen’s University, Kingston, ON, Canada

**Keywords:** extrapulmonary shunt, intrapulmonary vascular dilatations, luspatercept

## Abstract

A 70-year-old man with transfusion-dependent myelodysplastic syndrome was treated with luspatercept. Nine months into treatment and 2 months after dose increase, he developed new hypoxemia with a room air resting oxygen saturation of 88%. He was admitted, requiring up to 90% Fio_2_ by high-flow nasal cannula to maintain saturations ≥ 92%. CT scan demonstrated stable mild probable usual interstitial pneumonia, without ground-glass, pulmonary embolism, or arteriovenous malformation. Agitated saline contrast-enhanced echocardiogram was late positive, consistent with intrapulmonary shunt. Macroaggregated albumin (MAA) shunt scintigraphy demonstrated a shunt of 7.5%. Extensive investigations ruled out congenital portosystemic shunt and hepatopulmonary syndrome. We diagnosed hypoxemia from intrapulmonary vascular dilatation (IPVD), with luspatercept as a possible cause. Oxygen requirements improved, and supplemental oxygen was discontinued by 7 weeks after cessation. Repeat MAA 10 weeks after cessation confirmed shunt resolution (3.6% shunt). This case of reversible IPVD attributable to luspatercept highlights important monitoring implications in treated patients.

Intrapulmonary vascular dilatation (IPVD) is an uncommon cause of hypoxemia, most commonly seen in hepatopulmonary syndrome (HPS).^1^ Characterized by pulmonary precapillary and capillary dilatation, IPVDs in HPS may be caused by a loss of vascular tone, vascular remodeling, or true angiogenesis.^2^ IPVD is detected by agitated saline contrast-enhanced transthoracic echocardiography, whereby microbubbles are seen in the left atrium 3 to 6 heart cycles after appearance in the right atrium. Although common in patients with liver disease, only a minority have IPVD that is extensive or severe enough to cause hypoxemia.^3^

Luspatercept is a relatively new (US Food and Drug Administration-approved in 2019) modulating agent that increases erythropoietin effect, approved for anemia in adults with transfusion-dependent beta thalassemia and low- to intermediate-risk myelodysplastic syndrome (MDS).^4^ We describe a case of reversible IPVD in a patient treated with luspatercept for MDS.

## Case Report

A 70-year-old man with transfusion-dependent MDS with ring sideroblasts and multilineage dysplasia was treated with luspatercept 1 mg/kg every 3 weeks for 7 months, then increased to 1.75 mg/kg every 3 weeks. Two months later, on routine assessment, he was found to have asymptomatic hypoxemia with a room air resting oxygen saturation of 88%, decreasing to 78% on exertion. Historic baseline resting oxygen saturations were 96% to 100% a month before and at all assessments in the prior 3 years.

**Figure 1:**
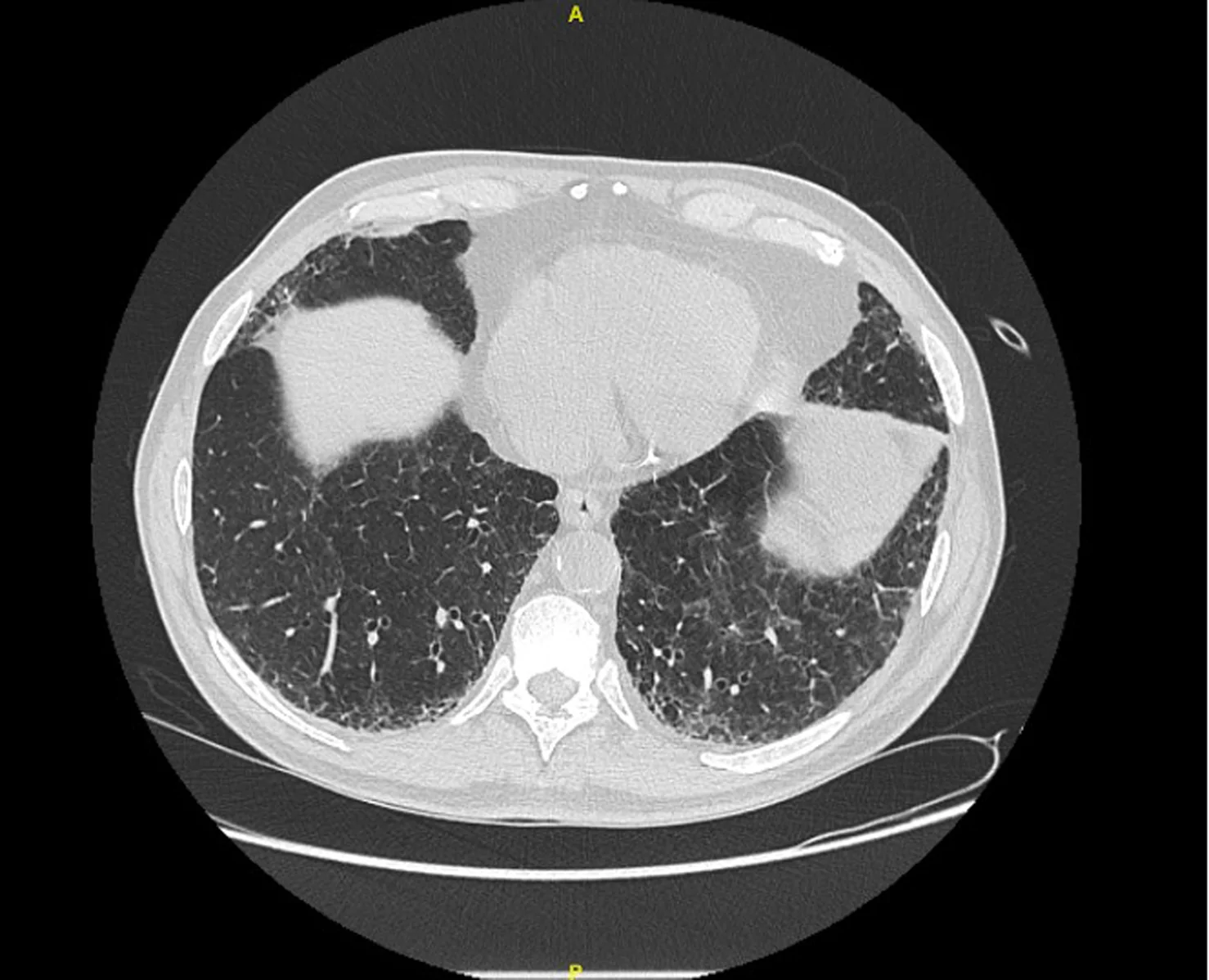
CT chest imaging demonstrating subpleural reticulation, traction bronchiectasis, and bronchiolectasis with basal predominance, stable from prior imaging, meeting criteria for probable usual interstitial pneumonia.^5^

He was admitted to the hospital and required up to 90% Fio_2_ by high-flow nasal cannula to maintain saturations ≥ 92%. CT pulmonary angiogram demonstrated mild, basal predominant subpleural reticulations with traction bronchiectasis (probable usual interstitial pneumonia pattern^5^) (Fig 1), unchanged from a CT 9 months prior. There were no new ground-glass changes, pulmonary embolism, or pulmonary arteriovenous malformations.

**Figure 2:**
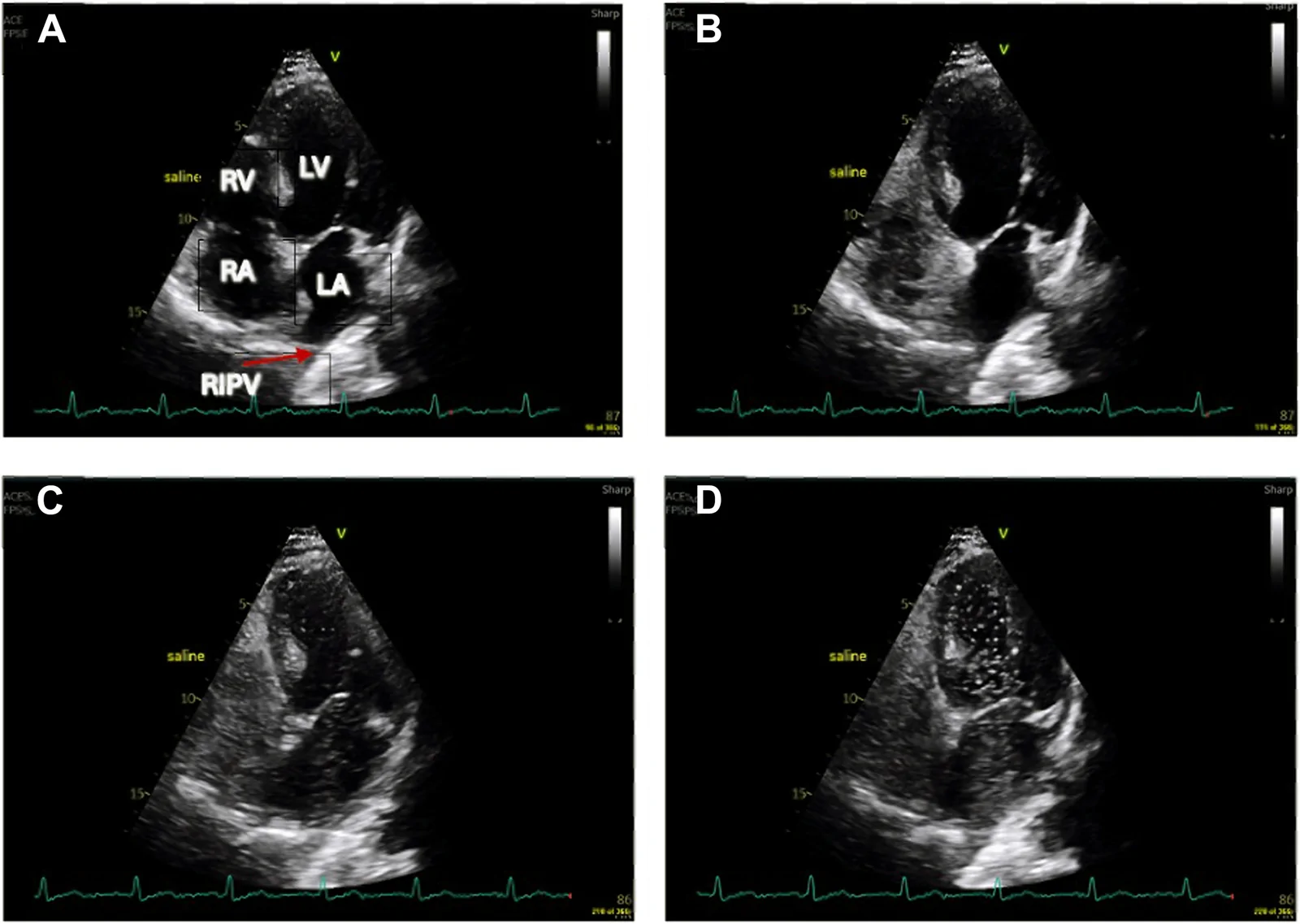
Sequential 4-chamber transthoracic echocardiography images of the patient with angulation for the pulmonary veins. A, Before saline echocontrast injection demonstrating anatomy. B, Beat 1 after injection appearance of echocontrast in the right side of the heart. C, Beat 4 after injection echocontrast appearing in the left atrium via the pulmonary vein. D, Beat 5 after injection opacification of the left side of the heart with endocardial definition suggesting significant shunt. LA = left atrium; LV = left ventricle; RA = right atrium; RIPV = right inferior pulmonary vein; RV = right ventricle.

Agitated saline contrast transthoracic echocardiogram demonstrated extensive echo contrast with endocardial definition in the left chambers after 5 cardiac cycles^6^ (grade 4) in keeping with significant intrapulmonary shunt (Fig 2). Transesophageal echocardiogram confirmed right-to-left shunt via all 4 pulmonary veins. No congenital cardiac abnormalities were identified. Macroaggregated albumin (MAA) shunt scintigraphy demonstrated a shunt fraction of 7.5% (normal, ≤ 6%). Ultrasound and MRI abdomen demonstrated no congenital portosystemic shunt; there was no cirrhosis or secondary signs of portal hypertension. Increased liver density suggestive of hemochromatosis prompted transjugular liver biopsy, which demonstrated hepatocellular iron deposition with minimal fibrosis, in keeping with prior transfusions. Liver enzymes and albumin were normal, international normalized ratio (INR) was 1.3, with mild bilirubin elevation (21 μmol/L). Transjugular hepatic angiogram demonstrated normal mean hepatic venous pressure gradient (4 mm Hg), ruling out HPS. A diagnosis of idiopathic IPVD was therefore considered. Given persistent oxygen dependency, lung transplant assessment was initiated.

**Figure 3:**
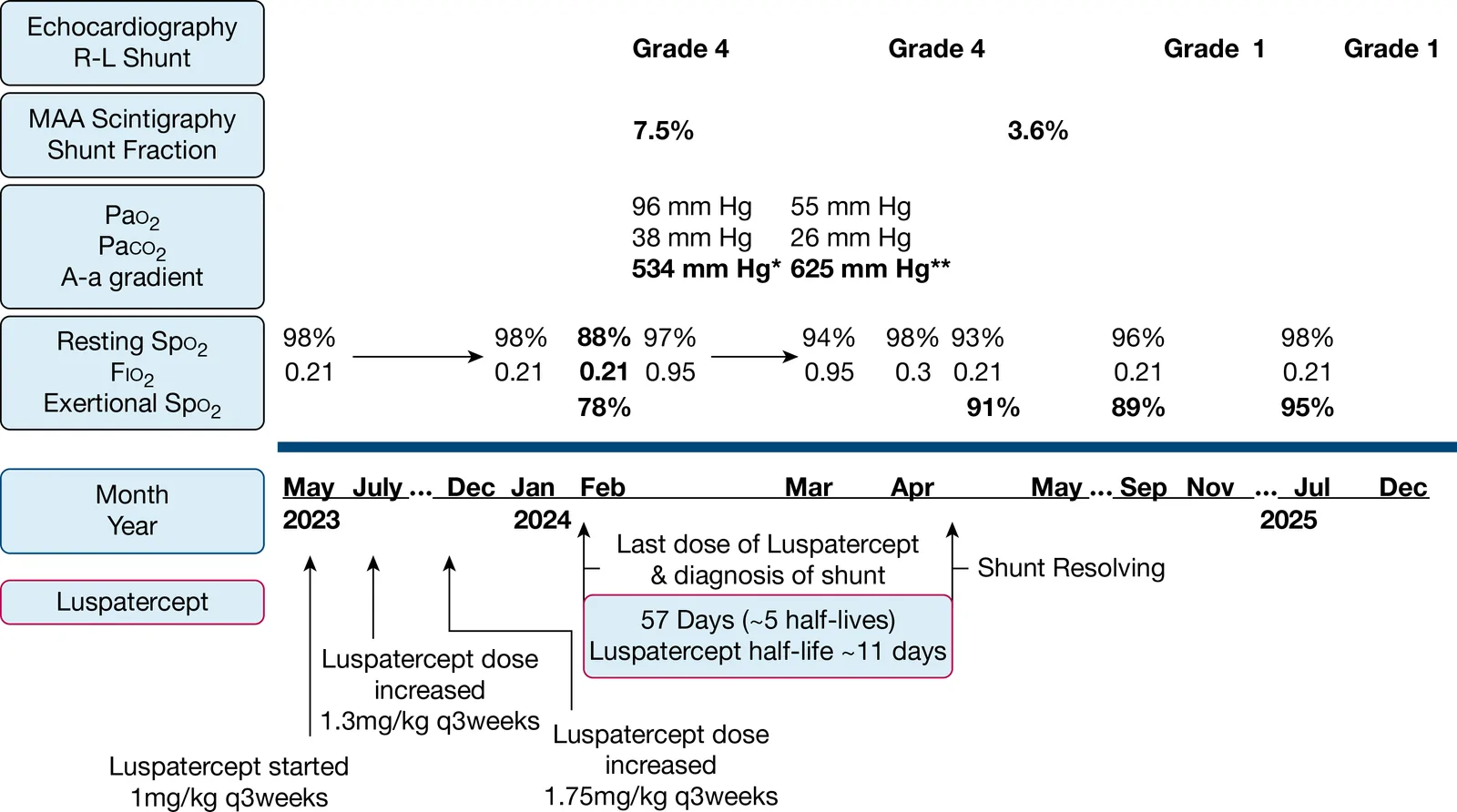
Chronological timeline of luspatercept treatment and clinical data. ∗Arterial blood gas performed on Fio_2_ 95%; ∗∗Arterial blood gas performed on Fio_2_ 100%. MAA = macroaggregated albumin; q3weeks, every 3 weeks; R-L, right-to-left; Spo_2_ = saturation of peripheral oxygen.

Luspatercept had been held at admission in the setting of acute illness, and given that the dose increase was the only recent therapeutic change, we suspended further use in case it was the cause. Six weeks after cessation, the patient’s oxygen requirements improved, and supplemental oxygen was discontinued by 7 weeks (Fig 3). Throughout this period, he remained asymptomatic from a respiratory perspective. Repeat MAA 10 weeks after cessation confirmed shunt resolution (3.6% shunt), at which point his oxygen saturations were 99% on room air at rest, decreasing to 91% on exertion during a 6-minute walk test. Repeat testing 15 months later demonstrated exertional oxygen saturations of 95% to 96%. A repeat agitated saline contrast transthoracic echocardiogram demonstrated reduction in the grade of intrapulmonary shunting to grade 1 (minimal shunt).^6^

## Discussion

Luspatercept inhibits 2 related pathways that normally preserve vascular endothelial integrity and promote endothelial quiescence^7^: (1) the transforming growth factor-beta (TGF-β) pathway, by acting as a “ligand trap” for TGF-β ligands (preventing SMAD2/3 signaling^4^); and (2) the bone morphogenetic protein (BMP) pathway, by trapping BMP10 (but not BMP9). Although circulating BMP9 levels are significantly reduced in humans with either HPS or portopulmonary hypertension,^8^ and loss of BMP9 signaling has been identified as a cause of IPVD in HPS,^9^ recent studies suggest that reductions in BMP10 may similarly contribute to IPVDs in HPS^8^, ^9^, ^10^ and also to arteriovenous malformations/telangiectasia in hereditary hemorrhagic telangiectasia.^11^ Susceptibility to derangements in BMP9/10 signaling may in turn be driven by loss-of-function mutations in GDF2, and its receptors ACVRL1 and ENG, while the variable nature of observed vascular phenotypic abnormalities may be mediated by host heterogeneity with respect to endothelial signaling, hemodynamics, and epigenetics.^12^ Additionally, emerging evidence suggests the role of both BMP9 and BMP10 in capillary network regulation, including pulmonary vascular remodeling.^13^ Meanwhile, sotatercept, a drug approved for pulmonary arterial hypertension, similarly acts as a ligand trap for selected TGF-β superfamily members^7^ and may lead to decreased circulating concentrations of BMP9 and BMP10.^14^^,^^15^ Not only has this drug been associated with vascular proliferation in the form of telangiectasias,^7^ but recent case series suggests that it can also cause IPVD^14^^,^^15^ and associated hypoxemia.^16^ Altogether, this suggests a possible mechanism for proliferative vascular disruption leading to IVPDs by luspatercept given its similar modulation of the BMP pathway.

Findings supportive of the diagnosis of IPVD in the patient include grade 4 delayed agitated saline contrast on echocardiogram, which is specific for intrapulmonary shunt, a pathologically elevated shunt fraction, and oxygen-responsive hypoxemia, which supports the presence of a precapillary shunt. Further supporting the role of luspatercept in the patient, hypoxemia was first noted after the third higher dose, when luspatercept would have reached steady state.^16^ Timing of oxygenation improvement at 6 weeks was also shortly after expected drug elimination (half-life, approximately 11 days).^16^ Although we did not measure BMP9/BMP10 levels in the patient and cannot exclude a genetic susceptibility to IPVD, the combination of the clinical findings suggestive of IVPD, the timing of hypoxemia and intrapulmonary shunt onset and resolution relative to luspatercept treatment, along with emerging evidence of similar phenomena described in patients treated with sotatercept, which has a similar mechanism of action, are all strongly suggestive of luspatercept as the causative agent.

In summary, we describe a case of reversible hypoxemia from IPVD secondary to luspatercept. The mechanism of action likely relates to luspatercept’s inhibition of TGF-β and BMP pathways and appears to be similar to emerging evidence related to sotatercept, suggesting a potential class effect of IPVD susceptibility related to these medications. This adverse reaction has important implications for monitoring of patients on this agent considering recently proposed recommendations that patients initiated on sotarcept receive a baseline and follow-up echocardiogram with agitated saline contrast to evaluate for the development of intrapulmonary shunting.^14^ Broader studies are required to further characterize the prevalence and clinical implications, and measurement of BMP9/10 levels should be considered in future cases to improve understanding of pathophysiologic processes.

## Financial/Nonfinancial Disclosures

None declared.

## References

[1] DuBrock H.M., Krowka M.J., Forde K.A. (2018). Clinical impact of intrapulmonary vascular dilatation in candidates for liver transplant. Chest.

[2] Zhao X., Kotha S., Nayyar D. (2023). Physiologic changes in the hepatopulmonary syndrome before and after liver transplant: a longitudinal and predictor analysis. Hepatology.

[3] Fallon M.B., Krowka M.J., Brown R.S. (2008). Impact of hepatopulmonary syndrome on quality of life and survival in liver transplant candidates. Gastroenterology.

[4] Fenaux P., Platzbecker U., Mufti G.J. (2020). Luspatercept in patients with lower-risk myelodysplastic syndromes. N Engl J Med.

[5] Raghu G., Remy-Jardin M., Myers J.L. (2018). Diagnosis of idiopathic pulmonary fibrosis: an official ATS/ERS/JRS/ALAT clinical practice guideline. Am J Respir Crit Care Med.

[6] Parra J.A., Bueno J., Zarauza J. (2010). Graded contrast echocardiography in pulmonary arteriovenous malformations. Eur Respir J.

[7] Hoeper M.M., Badesch D.B., Ghofrani H.A. (2023). Phase 3 trial of sotatercept for treatment of pulmonary arterial hypertension. N Engl J Med.

[8] Rochon E., Krowka M., Bartolome S. (2020). BMP9/10 in pulmonary vascular complications of liver disease. Am J Respir Crit Care Med.

[9] Robert F., Certain M.C., Baron A. (2024). Disrupted BMP-9 signaling impairs pulmonary vascular integrity in hepatopulmonary syndrome. Am J Respir Crit Care Med.

[10] Owen N.E., Nyimanu D., Kuc R.E. (2020). Reduced circulating BMP9 and BMP10 and elevated endoglin are associated with disease severity, decompensation and pulmonary vascular syndromes in patients with cirrhosis. EBioMedicine.

[11] Tillet E., Bailly S. (2015). Emerging roles of BMP9 and BMP10 in hereditary hemorrhagic telangiectasia. Front Genet.

[12] Sangam S., Yu P.B. (2024). Hepatopulmonary syndrome, another face of dysregulated BMP9 signaling. Am J Respir Crit Care Med.

[13] Bouvard C., Tu L., Rossi M. (2022). Different cardiovascular and pulmonary phenotypes for single- and double-knock-out mice deficient in BMP9 and BMP10. Cardiovasc Res.

[14] Mitchell S., Gomez-Rojas O., Mathavan A. (2025). Development of new intrapulmonary shunts in pulmonary arterial hypertension patients treated with sotatercept. Am J Respir Crit Care Med.

[15] Olsson K.M., Savale L., Guignabert C. (2025). Severe hypoxemia and pulmonary capillary dilatations in pulmonary arterial hypertension patients treated with sotatercept. Am J Respir Crit Care Med.

[16] Luspatercept (RX). Medscape drugs & diseases. Accessed April 22, 2025. Medscape website. https://reference.medscape.com/drug/reblozyl-luspatercept-1000359

